# Body Contouring Finesse: Dynamic Definition Liposculpture and Bipolar Radiofrequency Microneedling

**DOI:** 10.1093/asj/sjae152

**Published:** 2025-01-16

**Authors:** Alfredo E Hoyos, Mauricio E Perez Pachon, Neil M Vranis

## Abstract

Dynamic definition liposculpture (HD2) is considered a highly sought after procedure in body sculpting surgery by patients. Radiofrequency microneedling is a cutting edge technology with evidence-based outcomes demonstrating skin tightening and retraction. These ancillary procedures complement and enhance the results of dynamic definition liposculpture. A retrospective review of patient records from 2022 to 2024 was conducted. All patients who underwent high definition (HD) or HD2 in combination with fractional radiofrequency microneedling treatments by the senior author (A.E.H.) were included. Data collected included patient demographics, areas treated, and any complications. A total of 86 patients were included: 16 in 2022, 62 in 2023, and 8 in 2024. The most frequently treated area was the abdomen, followed by the back, face, neck, thighs, and arms. The average age of patients was 40.0 years in 2022, 40.8 years in 2023, and 44.4 years in 2024. The average BMI was 23.9 kg/m^2^ in 2022, 24.3 kg/m^2^ in 2023, and 25.2 kg/m^2^ in 2024. Minimal complications were observed, with some patients requiring further interventions such as scar correction and nevus resection. Avoiding superficial liposuction by relying on radiofrequency microneedling to target the adipose tissue directly beneath the dermis decreases the risk for iatrogenic (cannula related) superficial contour irregularities and makes the overall operation safer and more reliable.

Following the Industrial Revolution, the rise of technology has been exponential in all industries. Consequently, medicine, and in particular plastic surgeons, have relied on technologic devices and advances to improve the safety and outcomes of clinical practice. Recently, the power of controlled radiofrequency (RF) has been utilized in both microneedling and bipolar devices to target dermal and subdermal collagen disposition and structure. Evidence has shown that this promotes remodeling, tightening, and adipolysis to improve skin texture, thickness, and contour.^1,2^ Surprisingly, these devices have had a great impact on the general outcome after liposuction and moreover have become an integral component of dynamic definition liposculpture (HD2), previously described by the senior author (A.E.H.).^3,4^ This is an upgrade to the previously published high-definition (HD) liposculpture technique, which carves the superficial and intermediate adipose layers of the skin and creates controlled contours to achieve an athletic and natural body.^4^

Techniques for advanced liposculpture take advantage of different technologies that allow the surgeon to safely reshape a patient's torso and extremities based on their unique underlying anatomy. Up-to-date concepts in body sculpting finesse consider body phenotype and variable degrees of muscularization, and safe fat grafting practices have permitted a broader spectrum of possibilities for our patients such that we are now able to plan a procedure that suits not only the patient's anatomy but also satisfies their expectations.^5^ Throughout this supplemental article, we describe our experience in blending dynamic definition liposculpture with radiofrequency microneedling to optimize outcomes of patients undergoing body sculpting procedures.

## RADIOFREQUENCY MICRONEEDLING TECHNICAL ASPECTS

In the basic science and clinical literature, it has been reported that bipolar fractionated RF microneedling devices (Morpheus8, InMode, Lake Forest, CA) have decreased the amount of subdermal adipose tissue while tightening dermal and subdermal connective tissue through a process of remodeling and neocollagenesis.^1,2^ The operator can manipulate depth of microneedle penetration (1-7 mm of depth), the degree of energy delivered per pulse, and the number of pulses delivered to a certain area (pulse count/cm^2^).Targets include the epidermis, dermis, and subcutaneous adipose tissue. The particular device the senior author (A.E.H.) prefers in his practice comes with a choice of handpiece. The head of the handpiece contains 12, 24, or 40 sharp, stainless steel, gold-coated microneedles. These are selected based on anatomic area, thickness of dermis and subcutaneous tissue, and the magnitude of desired treatment effect. The goals of treatment include fat liquefaction, contraction of the reticular dermis, and remodeling of the connective tissue (fibroblast stimulation). The more laxity and the less adipose tissue, the less energy and depth are applied. Typical settings are, for the periorbital area, 2 mm, 15W to 30W; face, 3 to 4 mm, 25W to 40W; and body, 2 to 7 mm, 25W to 50W. Burst mode is preferred for HD due to the vulcanization technology that allows multiple depths in the same pulse, which creates cross links between long collagen fiber molecules in a stratified depth.^2^

## ARTISTIC ANATOMY

### Power vs Definition

**Figure 1.**
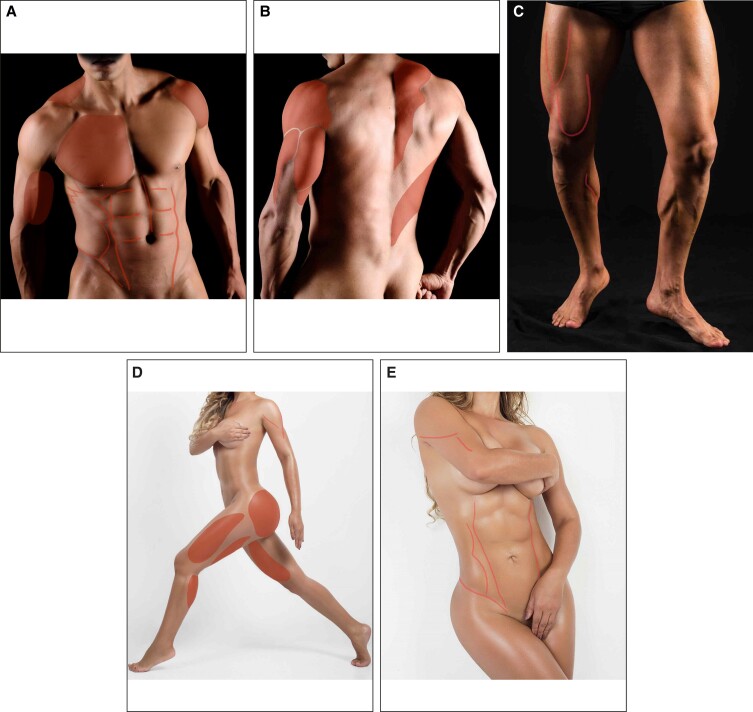
(A) Torso frontal oblique, (B) torso posterior oblique, and (C) lower extremity frontal oblique views of a 28-year-old athletic male model demonstrating cutaneous landmarks of muscle definition. (D) Semilateral and (E) oblique frontal views of a 26-year-old athletic female model. The orange shades are areas of muscular convexity and dotted lines are convex areas between major muscle groups.

Muscles are individual bundles of muscle fibers that share a common origin and insertion. The ratio of muscle size to overlying skin and subcutaneous thickness determines the degree of visible definition. A patient's physique can be altered by manipulating both factors. Removing subcutaneous adiposity and creating any degree of volume enhancement or projection with intramuscular or subcutaneous adipose grafts improves the overall muscular appearance. For HD2, we have divided the muscles according to gender (Figure 1). Definition muscles are those requiring sharp edges and demarcated limits to provide a “shredded” perception of the body. Certain muscles are considered “masculinizing” and would distort the delicate, soft, feminine appearance. These muscles include the deltoids, trapezius, triceps brachialis, gluteus medius, pectoralis major, latissimus dorsi, and serratus anterior.

### Feminizing Facets of the Torso

**Figure 2.**
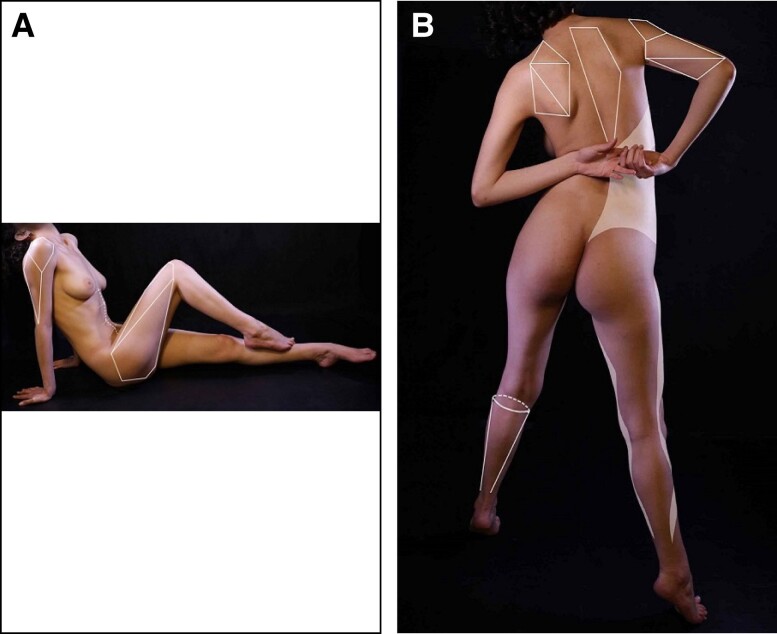
(A) Semilateral and (B) oblique frontal views of a 26-year-old athletic female model. The orange shades are areas of muscular convexity and dotted lines are convex areas between major muscle groups.

Feminizing facets of the torso are conceived as planes over different body segments that interact with each other to give the body a natural feminine silhouette (Figure 2). These facets differ from the sharp edges of male body sculpting, and can be found at the posterior arm; with shadowing here more of a delicate diamond shape. In the posterior torso the scapulae and trapezius muscle interact to form different planes. Laterally, in the waist area, the flanks act as a rhomboid wraparound with a smooth transition to the latissimus dorsi. Anteriorly, the rectus abdominis transitions from the rib cage with more shadowing laterally because the external oblique forms a gentle concavity.

## METHODS

We conducted a retrospective review of patient records from the last 3 years at a single center in Bogotá, Colombia. Our aim was to identify patients who underwent HD or HD2 in combination with bipolar fractionated RF microneedling treatments. All procedures were performed by the senior author (A.E.H.). Data collected included patient demographics, areas treated, and any noted complications. Specifically, we analyzed the distribution of treatment areas and documented the frequency of each area treated across the years 2022, 2023, and 2024.

### Preoperative Considerations

#### Patient Evaluation

The principles of the Declaration of Helsinki were followed, including obtaining written informed consent from all participants. Similar to any surgical procedure, the consultation included a thorough past medical history and a detailed physical examination to determine the patient's BMI, areas of excess adipose deposits, muscular structure, and body biotype. We preferred to utilize an abdominal wall ultrasound when scars were present over the abdominal wall or abdominal hernias were suspected. Patients were required to stop any tobacco products for at least 2 weeks before and 2 weeks after surgery. Heavy smokers or those experiencing cessation difficulties were referred to their primary care provider for more extensive cessation strategies.

Medical exclusion criteria for undergoing HD2 included patients with a high BMI (≥30 kg/m^2^), significant cardiovascular pathology requiring intervention or treatment, blood clotting disorders, active smokers, ASA III, a history of psychiatric disorders (eg, body dysmorphic disorder), and a hemoglobin ≤ 10 g/dL. Relative contraindications included an age greater than 65, autoimmune or immunodeficient disorders, and patients with preoperative hemoglobin levels between 10 and 12 g/dl.

#### Intraoperative Safety Protocols

HD liposculpting became extensively popular worldwide in the late 2000s and currently is considered a safe and effective technique for body contouring surgery. Strict protocols for patient preparation and intraoperative measures are in line with evidence-based medicine that have been customized for our practice. For thromboembolic event prevention, preoperative risk stratification is paramount. Calculating the Caprini score allows for modifiable risk factors to be addressed before surgery (ie, weight loss, regular exercise, quitting smoking, suspending oral contraceptives). Lower extremity compression socks, intermittent pneumatic compression devices, normothermia, early ambulation, and shortened operative times were well accepted interventions to reduce the risk of postoperative embolic events. For patients with Caprini scores of 3 or greater, chemoprophylaxis was usually administered postoperatively (Table 1).

**Table 1.** Protocol for Prevention of Thromboembolic Events

Protocol steps
1. Suspend OCPs and HRT 3 weeks before surgery and resume after 2 weeks.
2. Avoid prolonged periods of sitting 24-48 hours before surgery.
3. For patients whose travel time is ≥8 hours, delay surgery for 48-72 hours.
4. Use intermittent pneumatic compression boots during surgery.
5. Use compression stockings for 5-7 days after surgery.
6. Patient early mobilization, preferably first 4-6 hours after surgery.
7. Guide chemoprophylaxis by preoperative Caprini score.

HRT, hormone replacement therapy; OCPs, oral contraceptives.

Intraoperatively, we optimized hemostasis with epinephrine and tranexamic acid in the tumescent fluid in addition to careful hemostasis for incisional procedures. Constant communication with the anesthesiologist was maintained throughout the procedure to avoid hypothermia and perioperative hemodilution. We considered transfusion only if the hemoglobin was less than 9 g/dL and the patient was symptomatic, or if the hemoglobin dropped below 7 g/dL.

### Surgical Planning

#### Markings

**Figure 3.**
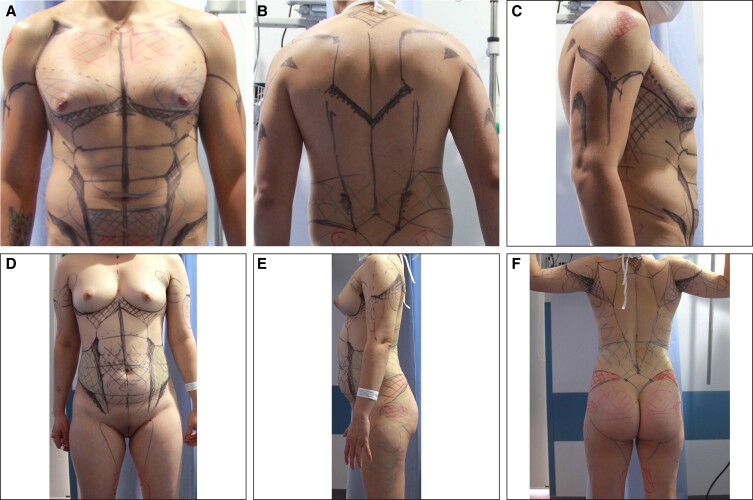
(A) Frontal, (B) posterior, and (C) lateral views of a 35-year-old male patient. (D) Frontal, (E) lateral, and (F) posterior views of a 29-year-old female patient. Black/blue lines are preoperative markings to delineate intersection of major muscle groups and will translate to lines of definition. Line thickness notes areas of widened negative contouring, and cross-hatched areas are defuse areas of liposuction debulking.

All markings were done with the patient in the standing position. Anatomic landmarks were outlined for reference, including negative spaces, forbidden areas (adhesion zones), fat deposits, transition and dynamic zones, and those for deep and superficial liposuction. Sharp muscular edges were etched for males, and curvilinear, gentle transitions were drawn for females (Figure 3). A color code that each surgeon was familiar with was utilized and is recommended; for example, green indicated negative spaces, red was for adhesion and transition zones, blue for fat deposits, black for anatomical landmarks, crossed lines for fat grafting, and so forth.

#### Degree of Muscular Definition

Aesthetic standards for muscular contours and definition differed by the individual's gender, identity, and body image preferences. Preoperative markings and intraoperative execution were tailored on a case by case basis. To perform these surgeries, we introduced a new variable of basic (B), moderate (M), or extreme (X) muscular definition.^4^ This was not only determined by the patient’s desire but also had to comply with the patient's body biotype and underlying musculoskeletal structure.^6,7^ To put this in clinical perspective, ectomorph patients could elect for any of the 3 degrees of muscular definition (BMX), but endomorph patients and mesomorph patients had to choose between 2 (BM and MX, respectively). This was because of the unnatural appearance that would result from an exaggerated deviation of a patient's underlying body type.^8,9, 10^

### Intraoperative Considerations

#### Hypothermia Prevention

Harmful effects following hypothermia include increased risk of surgical site infection, coagulopathy (platelet dysfunction), myocardial complications (arrhythmias and ischemia), prolonged recovery times, among others. Table 2 outlines the senior author’s (A.E.H.) protocols for maintaining normothermia during the procedure.

**Table 2.** Protocol for Hypothermia Prevention in High Definition Liposculpture (HD) and Dynamic Definition Liposculpture (HD2)

Protocol steps
1. Turn off the air conditioning inside the operating room (OR) before the patient enters.
2. One-hour patient prewarming with a Bair Hugger (3M, Saint Paul, Minnesota) before admission to the OR (warm air at 38°C/100.4 °F).
3. Insert esophageal thermometer (after endotracheal intubation) for continuous temperature monitoring.
4. Air conditioning inside the OR must be set to 22°C-23°C (68-71.6 °F).
5. Keep both intravenous fluids and tumescent solution for infiltration at 37.5°C (99.5 °F).^a^
6. Use the Blanketrol (Gentherm, Northville, MI) system during the entire procedure.^b^
7. Keep the surgical blankets as dry as possible: decrease non-sensible temperature loss.
8. Turn off the OR air conditioning about 30 minutes before the procedure termination.
9. Use the Bair Hugger (at 38°C/100.4°F) to keep the patient in normothermia after surgery.

^a^We use the ANOVA precision cooker (ANOVA Applied Electronics, Inc., San Francisco, CA) to keep fluids at this temperature in a bain-marie. ^b^Blanketrol is a temperature regulation system (computer-controlled heater + circulation pump + water-filled blanket) that is placed beneath the clothing of the surgical table.

#### Stealth Incisions

Minimal access incisions (5 mm) were placed over hidden areas or along skin creases to mitigate postoperative stigmata. The most common stealth incisions included the anterior axillary fold, posterior axillary fold, distal elbow, nipple (for male patients) and inframammary fold (female patients), umbilicus, inguinal crease, intergluteal crease, infragluteal crease, knee, popliteal, Achilles, lateral thigh, and posterior neck midline.

#### Tumescent Solution

A standard mixture of epinephrine, lidocaine, and tranexamic acid were added to lactated ringers, with 1 mg of epinephrine per liter of tumescent solution (ideal dose: 0.15 mg/kg). To prevent the non-sensible absorption of epinephrine, we did not exceed 9 mg of epinephrine in total. Lidocaine concentration peaked in the plasma an average of 12 to 24 hours after infiltration. It was therefore imperative to monitor patients for toxicity or complications for up to 24 hours after surgery. We abided by the standard maximum dose for lidocaine of 35 mg/kg. Patients remain in the recovery room for a minimum of 6 to 12 hours after surgery with clear instructions to watch for both lidocaine intoxication and pulmonary edema. Infiltration was allowed to sit for 5 to 10 minutes for an even solution distribution before starting emulsification (local vasoconstrictive effect).

#### Ultrasonic Emulsification

The operation began with emulsification of the superficial layer. Back and forth gentle movements of the probe were performed until resistance was decreased to minimal effort. A pulsed mode of 70% to 80% was set for the anterior and posterior torso (superficial layer) and a pulsed mode of 50% to 60% for the arms and thighs (both superficial and deep layers) and in young patients without fibrosis. The continuous mode of 70% to 80% was reserved for the deep layer in bulky areas over the anterior and posterior torso. The 3 endpoints for successful ultrasonic emulsification (VASER; Solta Medical–Bausch Health Companies Inc., Bothell, WA) treatment were (1) time, 1- to 2-minute maximum per 100 mL of infiltrated solution; (2) decreased or minimum tissue resistance; and (3) temperature, when the palpable tissue was warmer than the surgeon's hand.

#### Fat Extraction

**Figure 4.**
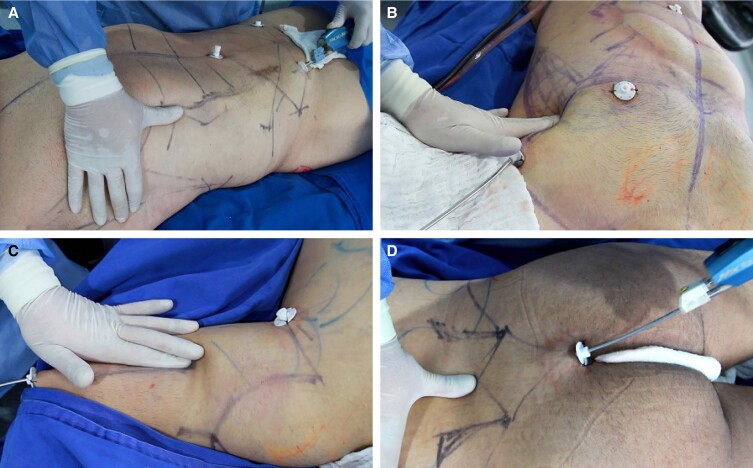
Intraoperative photographs of (A, B) abdomen, (C) flank, and (D) back liposuction of a 35-year-old male patient.

**Figure 5.**
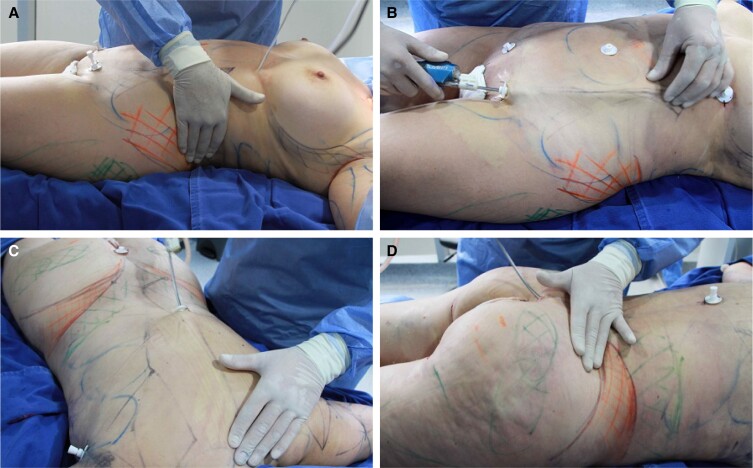
Intraoperative photographs of abdomen (A, B) abdomen, (C) back, and (D) flank liposuction of a 29-year-old female patient.

Surgeons prefer liposuction systems such as PowerX (Solta Medical–Bausch Health) and MicroAire power-assisted liposuction (MicroAire Surgical Instruments, Charlottesville, VA) to minimize operator fatigue. Suctioning the deep layer revealed the gross anatomy, and superficial layer liposuction unveiled the anatomical details and artistic landmarks (Figures 4, 5). In males we began with superficial liposuction of the definition lines with a small cannula (3 mm) to mitigate risk of contour irregularities and then slowly progressed to larger cannulas (4-5 mm) for deeper suctioning. In females focus was initially on creating gentle valleys and shadows with 3- to 4-mm cannulas. Once the negative spaces were defined, anatomic regions were evenly debulked to an endpoint determined by the surgeon.

#### Fat Transfer

The following tenets were created by the senior surgeon (A.E.H.) as a result of extensive experience and were routinely followed: (1) intramuscular fat grafts must be placed as superficial as possible; (2) applying the Mathes and Nahai vascular classification system, always direct the cannula perpendicular to the direction of the main muscle pedicle; and (3) access point entry should penetrate the muscle fascia as far as possible from the main pedicle.^11^ The most common large muscles that we have fat grafted for muscle augmentation are the deltoids, pectoralis major, biceps, trapezius, gluteus maximus, gluteus medius, vastus lateralis, and medial and lateral gastrocnemius.

After fat harvest with the power liposuction systems, lipoaspirate was decanted and washed. After fat processing, a peristaltic pump was utilized for EVL (expansion vibration lipofilling).^12^ This technique has been widely accepted as safe and efficacious for high-volume lipofilling. This was performed with a blunt-tip 3-mm cannula, injecting in a constant retrograde fashion. Aliquots of fat were deposited with a multiple-layer approach. The subcutaneous plane was compulsory at the gluteal region, and the intramuscular, subfascial plane was preferred for augmenting most power muscles.^13^

#### Microneedling Radiofrequency Application

A thorough preoperative assessment of the skin and subdermal thickness and its laxity through pinch tests was imperative to stratify patients. Patients with suboptimal inherent skin elasticity or thickness were indicated for simultaneous microneedling radiofrequency treatment in addition to the detailed liposuction procedure. For thin skin flaps the power variable was decreased, and we did not perform superficial burst shots. This mitigated the risk of thermal damage to the dermis and epidermis.

**Figure 6.**
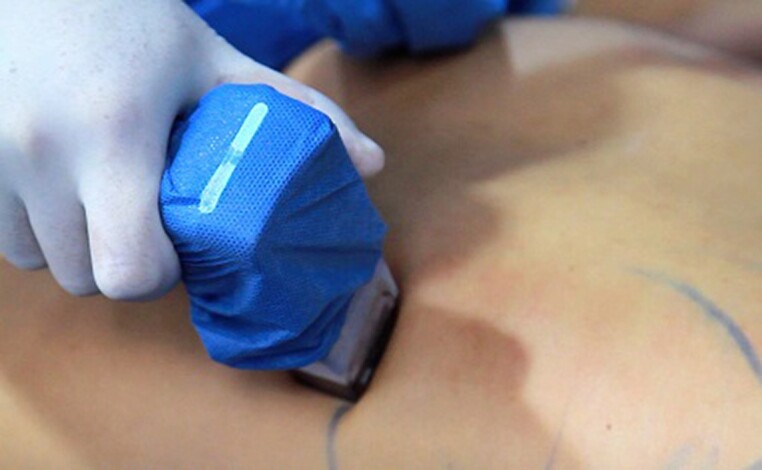
Intraoperative photograph depicting use of the Morpheus8 microneedling radiofrequency device after completion of liposuction in a 29-year-old female patient.

Meticulous technical execution also decreased risk of thermal injury. The handpiece must be firmly positioned over the treatment area and make complete contact with the skin (Figure 6). Complete contact of the head of the handpiece completes the circuit that is critical to maintaining the bipolar nature of the device. Once the handpiece was positioned appropriately, with approximately 30% overlap with the previous area, the pedal was pressed to deliver the RF energy. The operator could choose to toggle between a single press-release for the cycle mode or continuous pressing of the pedal for the fixed and burst mode (preferred).

**Figure 7.**
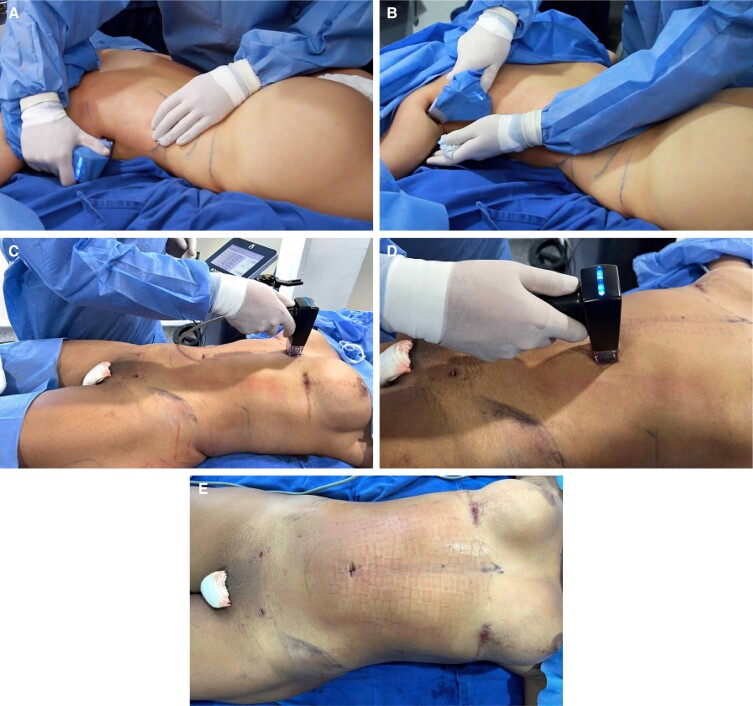
Intraoperative photograph of a 29-year-old female patient depicting use of the Morpheus8 microneedling radiofrequency device on the (A, B) flanks and (C, D) abdomen after completion of liposuction. (E) The “grid” pattern of the microneedling is expected after completion of the microneedling RF treatment.

We began this portion of the procedure over the zones where more flaccidity was seen. Typically this involved the proximal posterior arm, lower abdomen, top lateral posterior torso, medial thigh, and infragluteal region (Figure 7). Next, the negative spaces and the muscle limits of definition muscles were targeted to accentuate transition muscle zones. Delivery of RF energy was avoided over the muscles in which fat transfer was performed. We usually left some space in between shots to fill with a new one with a different depth or energy. The depth and energy must be customized to the patient's skin quality and subdermal thickness. The following settings were frequently chosen and can serve as reference points for the reader:

- Arms and thighs: 30W to 35W power, starting at a 6-mm depth, then 4 mm and 2 mm.
- Torso: Burst mode at 40W power, starting at a 7-mm depth, then 5 mm and 3 mm.
- Submandibular region: 50W, 40W, and 30W at 4 mm, 3mm, 2mm.

### Postoperative Considerations

**Figure 8.**
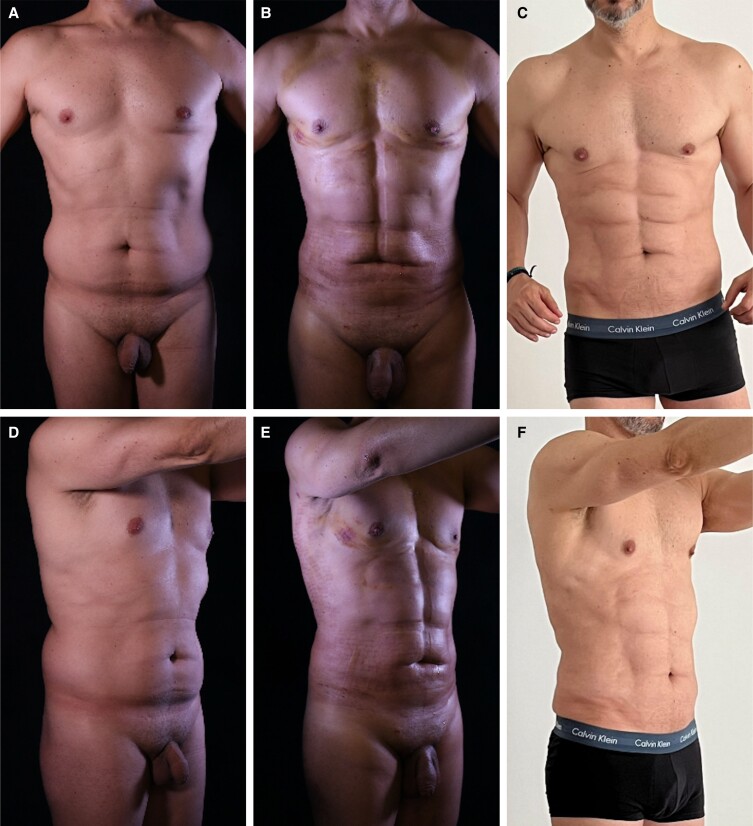
A 47-year-old male underwent high-definition liposuction combined with microneedling radiofrequency (RF) of the abdomen. (A, D) Based on the skin sagging in the lower abdomen, this patient might typically be considered for a miniabdominoplasty. However, the combination of high-definition liposuction with intraoperative microneedling RF improved skin retraction and eliminated the need for lipectomy. This improvement can be seen in (B, E) the early 48-hour photographs and (C, F) the final result 6-month postoperatively, after 2 additional sessions of RF.

**Figure 9.**
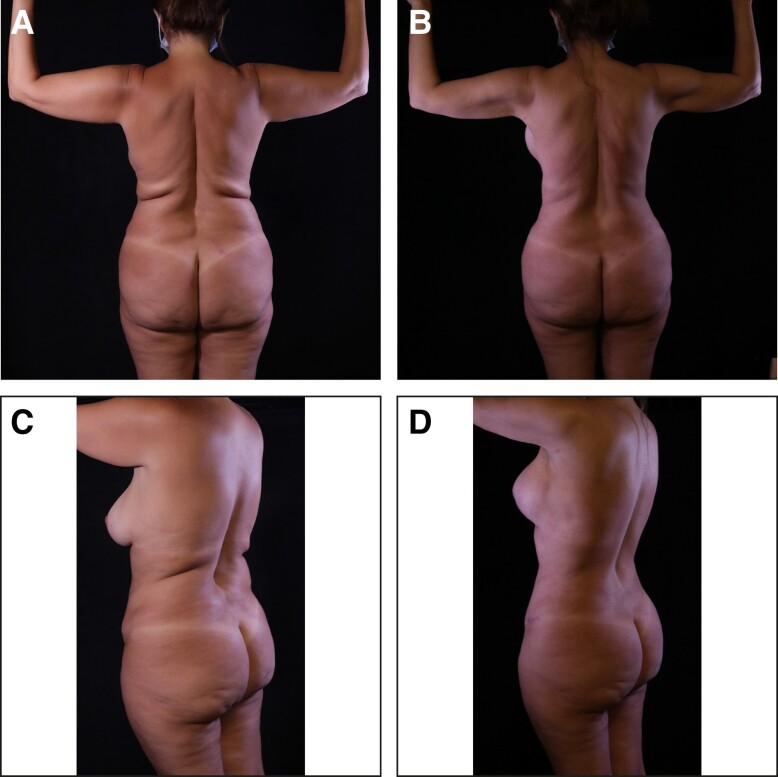
A 42-year-old female who underwent high-definition liposuction (HDL) and intraoperative microneedling radiofrequency (RF) of the arms and the back. Both HDL and RF helped improve the skin tightening of the arms and the posterior torso by almost disappearing the back rolls and improving the slim definition of the arms. This is well appreciated by comparison of (A, C) the preoperative photographs with (B, D) the 6-month follow-up postoperative photographs.

**Figure 10.**
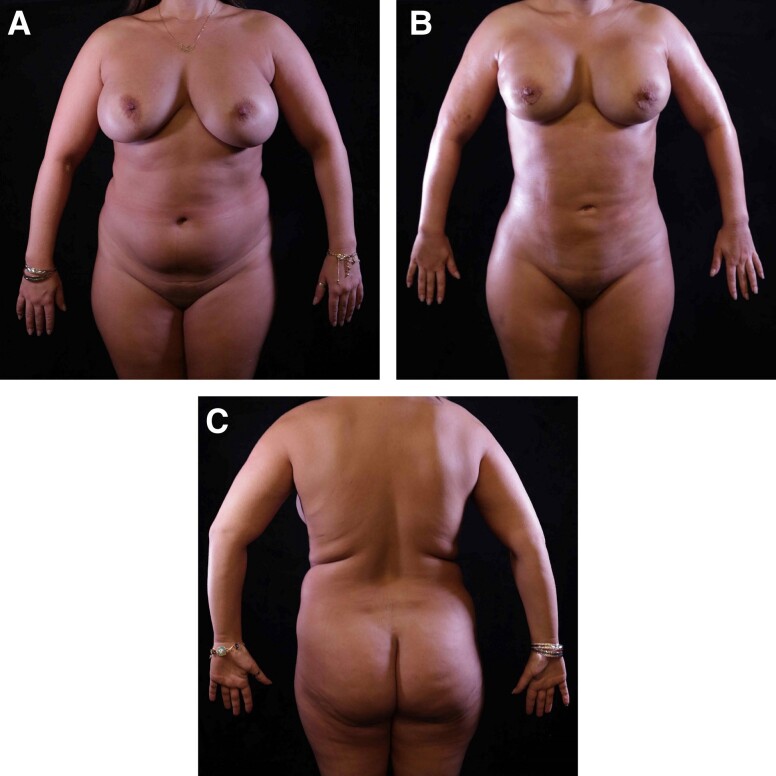
A 46-year-old female who underwent high-definition liposuction (HDL) and intraoperative microneedling radiofrequency (RF) of the arms and the back. Both HDL and RF helped improve the skin tightening of the arms, abdomen, flanks, and posterior torso. Note the improvement in back rolls, abdominal definition, and slim definition of the arms. This is well appreciated by comparison of (A, C) the preoperative photographs with (B, D) the 6-month follow-up postoperative photographs.

**Figure 11.**
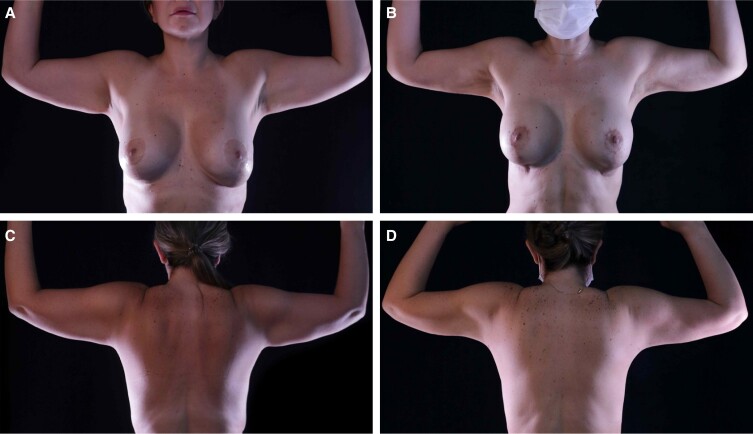
A 39-year-old female who underwent high-definition liposuction (HDL) and intraoperative microneedling radiofrequency (RF) of the arms and the back. Both HDL and RF helped improve the skin tightening of the arms. Circumferential arm reduction and improved definition can be appreciated by comparison of (A, C) the preoperative photographs with (B, D) the 6-month follow-up postoperative photographs.

Patients were closely monitored after surgery, with the first appointment usually 24 to 48 hours after surgery and then at 1 week, 1 month, 3 to 6 months, and 1 to 2 years. Results may take up to 6 months to be completely appreciated given the time it takes for the edema to resolve, the fat transfer to remain viable, and neocollagenesis to occur (Figures 8-11). Patients are advised to apply cooling to the treated area to reduce erythema and discomfort. Topical antibiotic and anti-inflammatory ointments are applied to incision sites or areas of potential thermal injury. Overall this will prevent local contamination and decrease swelling. Hyperbaric oxygen therapy is recommended for patients with flap ischemia or altered perfusion over any body area, usually 24 to 72 hours after surgery. Skin moisturizers (body and face lotions) and sunscreen may be started after 24 hours. Manual lymphatic drainage begins 24 hours after surgery, and external ultrasound therapy and pressotherapy are provided 24 to 48 hours after surgery. This is avoided in areas where fat grafting was performed.

Additional external RF can be performed 10 to 15 days after surgery to promote second-phase healing. Additional sessions—up to 3, 1 month apart—of radiofrequency microneedling can be performed in the office under topical anesthesia if additional skin tightening is required.

## RESULTS

A total of 86 patients underwent HD and HD2 in combination with fractional bipolar RF microneedling over the 3-year period from 2022 to 2024. The cohort included 16 patients in 2022, 62 patients in 2023, and 8 patients in 2024. The distribution of patients across these years accounted for 18.6%, 72.1%, and 9.3% of the total, respectively. The average age of patients was 40.0 years in 2022, 40.8 years in 2023, and 44.4 years in 2024. The interquartile range (IQR) for age showed variability, with 13.8 years in 2022, 9.5 years in 2023, and 17.5 years in 2024. The average BMI of patients slightly varied across the years, being 23.9 kg/m^2^ in 2022, 24.3 kg/m^2^ in 2023, and 25.2 kg/m^2^ in 2024, with respective IQRs of 3.3 kg/m^2^, 3.5 kg/m^2^, and 1.9 kg/m^2^. The weight of patients averaged 64.9 kg in 2022, 66.5 kg in 2023, and 70.0 kg in 2024, with weight ranges showing considerable variability. The average volume of lipoaspirate was 3346.4 mL in 2022, 3227.6 mL in 2023, and 3793.8 mL in 2024, with IQRs indicating a broad range of lipoaspirate volumes across the years (Table 3). Complications observed during the study period included a single thermal injury on the right arm immediately after ultrasound-based liposuction in 1 patient, and 1 patient with a mild asymmetry, none of them related to the combination of treatments but rather isolated events. These findings indicate a consistent application of the procedure with notable variations in patient demographics and treatment outcomes over the study period, alongside some instances of postprocedure complications that required additional medical attention.

**Table 3.** Patient Demographics

Year	2024	2023	2022
No. of patients	8	62	16
Male	1	54	13
Female	7	8	3
Percentage of total	9.3	72.1	18.6
Average age (years)	44.4	40.8	40.0
Age IQR	17.5	9.5	13.8
Age range	38.0	35.0	26.0
Average BMI (kg/m^2^)	25.2	24.3	23.9
BMI IQR	1.9	3.5	3.3
BMI range	3.6	11.1	10.3
Average weight (kg)	70.0	66.5	64.9
Weight IQR	15.0	15.5	13.0
Weight range	33	62	43
Average lipoaspirate (mL)	3793.8	3227.6	3346.4
Lipoaspirate IQR	2112.5	3247.5	2725.0
Lipoaspirate range	5600	6100	6200
Area treated (*n*)			
Abdomen	3	15	16
Back	2	10	8
Face	1	9	8
Neck	1	5	6
Thighs	1	6	3
Arms	3	5	0
Other areas	2	12	2

BMI, body mass index; IQR, interquartile range.

## DISCUSSION

Bipolar fractional RF microneedling has gained significant attention in recent years as a versatile and effective treatment modality for skin tightening and rejuvenation. When combined with HD or HD2 liposculpture, as seen in our study, it enhances the overall aesthetic outcomes by promoting skin contraction and improving the texture and tone of the treated areas.

Our retrospective cohort shows the efficacy and safety of this combined approach. The results indicated consistent application and positive outcomes across a diverse patient population. In our series, the most frequent treatment areas were the abdomen, followed by the back, face, neck, thighs, and arms. This distribution aligned with the common anatomical regions in which skin laxity and contour irregularities are most noticeable. Bipolar fractional RF microneedling in these areas facilitated significant skin retraction, which was crucial for achieving the desired definition and contouring in liposculpture procedures. Moreover, it seemed it might be helpful to move to more conservative treatments for patients with mild to moderate skin laxity. The average BMI and weight also showed variability, indicating that the combined treatment was suitable for patients with different body compositions. Complications were usually not associated with the combination of treatments but rather the result of each procedure alone. These findings underscored the importance of meticulous technique and careful patient selection to minimize adverse outcomes. The low complication rate also highlighted the safety of combining bipolar fractional RF microneedling with liposculpture. This adjunct technology has proven to be an effective adjunctive treatment in body contouring surgeries. Its ability to enhance skin tightening and improve surface texture complements the fat reduction and contouring achieved through liposculpture. The synergistic effect of these treatments results in superior aesthetic outcomes, with high patient satisfaction and minimal downtime.

### Limitations

Our study had several limitations. The retrospective design limited causal inferences, and the data relied on potentially incomplete patient records. With the study conducted at a single center with all procedures performed by 1 surgeon, the findings may not be generalizable. The small sample size, especially in 2024, affects statistical robustness. Variable follow-up periods and potential patient selection bias also limited the study. Future larger, multicenter, prospective studies are needed to validate these findings.

## CONCLUSIONS

Bipolar fractional RF microneedling treatments are increasingly popular as stand-alone therapies for skin tightening or in conjunction with other procedures to augment the surgical result. We have found it a worthy adjunct to HD and HD2 lipo-contouring procedures in patients who require additional skin tightening or dermal thickening. Surgeon must be aware of safe settings and carefully select patient indications to maximize outcomes and mitigate complications.

## References

[1] Dayan E , Burns AJ, Rohrich RJ, Theodorou S. The use of radiofrequency in aesthetic surgery. Plast Reconstr Surg Glob Open. 2020;8(8):e2861. Doi: 10.1097/GOX.0000000000002861 32983755 PMC7489578

[2] Dayan E , Chia C, Burns AJ, Theodorou S. Adjustable depth fractional radiofrequency combined with bipolar radiofrequency: a minimally invasive combination treatment for skin laxity. Aesthet Surg J. 2019;39(Supplement_3):S112–S119. Doi: 10.1093/asj/sjz055 30958550 PMC6460431

[3] Hoyos AE , Prendergast PM. The human form as art: contours, proportions, and aesthetic ideals. In: High-Definition Body Sculpting. Springer Berlin Heidelberg; 2014:3–18.

[4] Hoyos AE , Prendergast PM. High definition body sculpting. In: Art and Advanced Lipoplasty Techniques. GEOTAR Media; 2020:256.

[5] Paul M , Mulholland RS. A new approach for adipose tissue treatment and body contouring using radiofrequency-assisted liposuction. Aesthetic Plast Surg. 2009;33(5):687–694. Doi: 10.1007/s00266-009-9342-z 19543679 PMC2758217

[6] Vranis NM , Steinbrech D. Gluteal augmentation in men. Clin Plastic Surg. 2023;50(4):615–628. doi: 10.1016/j.cps.2023.05.003 37704328

[7] Stevens SS , Tucker WB. The varieties of human physique: an introduction to constitutional psychology. Am J Psychol. 1940;54(3):457–459. doi: 10.2307/1417706

[8] Zarins U , Kondrats S. Anatomy for Sculptors: Understanding the Human Figure. Exonicus. LLC; 2019.

[9] Simblet S , Davis J. Anatomy for the Artist. DK Publishing Inc; 2021.

[10] Hoyos AE , Perez ME, Domínguez-Millán R. Variable sculpting in dynamic definition body contouring: procedure selection and management algorithm. Aesthet Surg J. 2021;41(3):318–332. doi: 10.1093/asj/sjaa133 32455430

[11] Mathes SJ , Nahai F. Classification of vascular anatomy of muscles: experimental and clinical correlation. Plast Reconstr Surg. 1981;67(2):177–187. doi: 10.1097/00006534-198167020-00007 7465666

[12] Del Vecchio D , Wall S. Expansion vibration lipofilling: a new technique in large-volume fat transplantation. Plast Reconstr Surg. 2018;141(5):639e–649e. doi: 10.1097/PRS.0000000000004338 29465484

[13] Hoyos AE , Prendergast PM. Fat anatomy, metabolism, and principles of grafting. In: High-Definition Body Sculpting. Springer Berlin Heidelberg; 2014:83–91.

